# Food choice determinants and perceptions of healthy eating and food safety among online food delivery application users in Saudi Arabia: a cross-sectional study

**DOI:** 10.3389/fnut.2026.1887881

**Published:** 2026-08-27

**Authors:** Nawaf W. Alruwaili, Abdulhmed Alharbi

**Affiliations:** 1 Department of Community Health Sciences, College of Applied Medical Sciences, King Saud University, Riyadh, Saudi Arabia; 2 Department of Clinical Nutrition, King Abdullah Specialized Hospital, Ministry of National Guard Health Affairs, Qassim, Saudi Arabia

**Keywords:** convenience food, dietary behavior, food choice, food safety, intention–behavior gap, nutritional epidemiology, online food delivery applications, Saudi Arabia

## Abstract

**Background:**

Online food delivery applications (OFDAs) have fundamentally transformed dietary consumption patterns globally, yet their associations with food choice determinants, healthy-eating perceptions, and food safety concerns in Saudi Arabia remain incompletely characterized.

**Objectives:**

This study aimed to evaluate OFDA usage patterns and their sociodemographic determinants; identify factors influencing food choices; assess perceptions of healthy food availability and food safety within OFDAs; and determine whether usage frequency was associated with body mass index (BMI) or dietary healthfulness.

**Methods:**

A cross-sectional, online survey was conducted from February to April 2025 among Saudi residents aged ≥ 20 years who had used an OFDA at least once per month (*n* = 303). Sociodemographic associations with usage frequency were evaluated using chi-square tests; Mann–Whitney *U* and Kruskal–Wallis *H* tests assessed differences in perception across subgroups. Statistical significance was set at *p* < 0.05.

**Results:**

The sample was predominantly female (73.2%) and aged 20–29 years (70.5%), with 51.7% classified as frequent users (≥ 2–3 times/week). Usage frequency was significantly associated with region (*p* = 0.033) and monthly income (*p* = 0.023). Price (82.8%), food appearance (56.6%), and delivery timing (49.7%) were the primary determinants of food choice. HungerStation was the most-used platform (61.6%), followed by Jahez (53.3%). Despite 58.9% of participants reporting health-seeking intentions, fast food was the predominant choice (78.5%), revealing a prominent intention–behavior gap. Frequent OFDA users were more likely to report difficulty finding healthy options (*p* = 0.024), changed eating habits (*p* < 0.001), and increased appetite (*p* = 0.020). The overall food safety perception mean score was 3.90/5.00, with near-universal support for mandatory in-app displays of hygiene ratings (mean 4.56 ± 0.72). No significant associations were found between OFDA usage frequency and BMI (*p* = 0.659) or dietary healthfulness (*p* = 1.000).

**Conclusion:**

OFDAs are predominantly used to purchase energy-dense, nutrient-poor foods in Saudi Arabia, driven chiefly by price and convenience. Regulatory mandates for nutritional labeling, visibility of hygiene ratings, and algorithmic promotion of healthy options on OFDA platforms are urgently needed to improve population-level dietary behaviors.

## 1 Introduction

The global integration of smartphones into everyday life has fundamentally reshaped how individuals access, select, and consume food. Digital food retail environments—encompassing websites, mobile applications, and aggregator platforms—now constitute a primary interface between consumers and the food supply, providing unprecedented access to both products and nutrition-related information (1, 2). Online food delivery applications (OFDAs) allow users to browse menus, place orders, and receive restaurant-prepared meals at preferred locations, thereby decoupling eating from cooking and dramatically expanding the temporal and spatial reach of food retail. Between 2019 and 2022, the OFDA user base expanded by approximately 75%, reaching over 1.85 billion users globally (3). The COVID-19 pandemic served as a powerful accelerant for this adoption, as lockdown restrictions, closure of dining establishments, and heightened health anxieties collectively drove consumers toward home-delivery services.

In Saudi Arabia, OFDA adoption has been particularly pronounced and demographically concentrated. The domestic market was projected to surpass US$2 billion by 2024, with over 8 million active users (4). HungerStation, established in 2012, and Jahez have achieved dominant market positions, while newer entrants, including Keeta, Ninja, and To You, continue to expand their presence across all five geographic regions of the Kingdom (5). This expansion unfolds against a backdrop of profound dietary transition: rapid urbanization, sedentary lifestyles, declining household cooking practices, and the proliferation of fast-food culture have collectively contributed to rising obesity prevalence—now exceeding 35% in some adult demographic groups—and escalating rates of non-communicable diseases, including type 2 diabetes and cardiovascular disease (6).

From a public health perspective, OFDAs raise substantive concerns about dietary quality. Research consistently demonstrates that meals offered through delivery platforms tend to be high in saturated fats, sodium, and refined carbohydrates, while nutritious alternatives are under-represented and disproportionately expensive (7–9). Studies conducted in New Zealand, the United Arab Emirates, Kuwait, and Malaysia corroborate that the structural design of OFDA menus—dominated by fast-food listings, value-meal promotions, and visually compelling images of energy-dense items—actively steers users toward unhealthy selections (9–12). A particularly salient pattern, termed the “healthy-eating intention–behavior gap,” has been documented, in which users express a desire to eat healthily yet routinely select nutritionally poor options, citing limited availability and cost as barriers (12). Whether this gap is equally prevalent among Saudi consumers and which sociodemographic factors moderate it have not been systematically examined.

Beyond dietary quality, food safety represents a second critical public health dimension of OFDA use. The separation of food preparation from delivery creates novel hygiene risks related to temperature maintenance during transit, packaging integrity, and delivery-personnel sanitation practices (13, 14). Consumer awareness of these risks has increased—accelerated by the COVID-19 pandemic—yet access to systematic hygiene ratings and sanitation transparency within Saudi delivery platforms remains limited. The regulatory framework governing OFDA food safety standards in Saudi Arabia is still evolving, and consumer perceptions of safety and hygiene within these platforms have not been comprehensively described.

As of the survey period (February–April 2025), Saudi Arabia’s regulatory framework governing OFDA-related nutrition and food safety disclosure was at an early stage of implementation. The Saudi Food and Drug Authority (SFDA) began phased enforcement of mandatory allergen and calorie labeling for restaurants and food delivery applications only in November 2024, shortly before data collection for the present study began; more comprehensive measures—including mandatory caffeine-content disclosure, high-salt warning labels, and physical activity calorie-burn equivalents on both physical and digital menus—were not enforced until 1 July 2025, several months after data collection had concluded (15). Delivery-specific hygiene requirements, including mandatory identification for delivery personnel and formal registration of delivery operations under the national Food System Violations Table, were likewise introduced only in August 2025 (16). The OFDA platforms used by participants in the present study, therefore, largely lacked the structured nutritional and hygiene information that Saudi regulators have since mandated.

The present study therefore addressed these gaps by: (i) characterizing the frequency and sociodemographic patterns of OFDA use across Saudi regions; (ii) identifying the primary determinants of food choice among OFDA users; (iii) evaluating perceptions of healthy food availability and food safety within OFDAs; (iv) examining how sociodemographic factors modulate these perceptions; and (v) assessing whether OFDA usage frequency is independently associated with BMI or healthfulness of food choices. The findings are intended to inform evidence-based policy recommendations for the regulation and redesign of OFDA platforms to promote healthier dietary choices at the population level.

## 2 Materials and methods

### 2.1 Study design and ethical approval

A cross-sectional, online observational study was conducted in Saudi Arabia from February to April 2025. The study was designed and reported in accordance with the Strengthening the Reporting of Observational Studies in Epidemiology (STROBE) guidelines for cross-sectional research (see Supplementary Material 2). Ethical approval was granted by the King Saud University Standing Committee on Research Ethics (Reference: KSU-HE-25-094; approved on 11 February 2025). All participants voluntarily participated and provided verbal informed consent prior to completing the survey. The study’s objectives, the voluntary nature of the survey, and the confidentiality of responses were clearly explained in the survey introduction. No personally identifying information was collected.

### 2.2 Participants and sampling strategy

Eligible participants were: (i) aged ≥ 20 years; (ii) residing in Saudi Arabia at the time of data collection; and (iii) having used at least one OFDA at least once within the previous month. Individuals who had never used an OFDA were excluded. This minimum-frequency threshold was adopted directly from the inclusion criteria used by Saleh et al. (10) in their UAE-based adolescent OFDA study, ensuring methodological consistency with the comparable published literature upon which the present survey instrument was based, while excluding non-users for whom the perception items would not be meaningfully applicable. Both convenience and snowball sampling methods were employed to maximize geographic and demographic coverage. For analytical purposes, participants’ regions of residence were classified by aggregating Saudi Arabia’s 13 official administrative provinces into five conventional macro-regions (Central, Western, Eastern, Southern, and Northern), a grouping strategy widely adopted in Saudi epidemiological survey research to ensure adequate subgroup sample sizes for statistical comparison while preserving meaningful geographic and cultural distinctions (17). The survey was distributed via social media platforms (Facebook, X [formerly Twitter], Instagram, and LinkedIn) using a hyperlinked survey invitation. The invitation was disseminated organically through unpaid, text-based posts shared within the research team’s personal, professional, and university networks, accompanied by a brief description of the study’s aims, eligibility criteria, and a clickable survey link; no infographics, paid advertisements, or boosted posts were used. Respondents were encouraged to forward the survey link to eligible contacts, consistent with snowball recruitment. To enhance data validity in this self-administered online format, the survey platform employed forced-response logic requiring all items to be completed before submission, and an embedded screening question that immediately terminated the survey for respondents who reported no past-month OFDA use. Incomplete and duplicate entries were excluded prior to analysis. While these measures cannot fully replicate the validation safeguards of interviewer-administered data collection, they are consistent with quality-control practices reported in comparable OFDA survey studies using the same distribution method (10, 18). Of 492 individuals initially contacted, 166 declined participation, leaving 325 potential respondents; 22 were subsequently excluded for failing to meet the OFDA-use eligibility criterion (Supplementary Figure 1), yielding a final analytic sample of *n* = 303.

Sample size was estimated *a priori* following World Health Organization recommendations for prevalence studies (19), applying a 95% confidence interval, a standard deviation of 0.5, and a margin of error of 5%, yielding a minimum required sample of 300 participants. The sample obtained (303) exceeded this threshold.

### 2.3 Survey instrument

The questionnaire (see Supplementary Material 1) was adapted from validated instruments previously employed in comparable OFDA studies in the UAE and Kuwait (11, 18, 20) and comprised 23 structured, closed-ended questions organized across four sections. Section “1 Introduction” captured sociodemographic characteristics: biological sex, age, nationality, marital status, self-reported height (cm) and weight (kg), educational attainment, employment status, region of Saudi Arabia, living situation, and monthly income. Section “2 Materials and methods” assessed OFDA usage behaviors: frequency of use, applications utilized (multiple-response), cuisine types ordered (multiple-response), food-choice determinants (multiple-response), and whether participants actively sought healthy options when ordering (binary yes/no). Section “3 Results” contained seven statements (A1–A7) assessing perceptions of healthy food availability and the influence of OFDAs on dietary habits, rated on a five-point Likert scale (1 = strongly disagree to 5 = strongly agree). Section “4 Discussion” contained seven statements (B1–B7) assessing perceptions of food safety and delivery hygiene, rated on the same Likert scale. The complete questionnaire with response options is provided in Supplementary Material 1. Internal consistency of the two seven-item Likert subscales was assessed *post hoc* using Cronbach’s alpha computed from the present dataset: α = 0.622 for the healthy food perception subscale (A1–A7) and α = 0.638 for the food safety and hygiene perception subscale (B1–B7), both within the questionable-to-acceptable range conventionally reported for short multidimensional attitude subscales; reliability for the combined 14-item instrument was higher (α = 0.729), indicating acceptable overall internal consistency. These items were adapted from instruments with established content and face validity in previously published UAE and Kuwaiti OFDA studies (10, 11, 20), although formal psychometric validation (e.g., confirmatory factor analysis) of the adapted instrument itself had not been performed prior to fielding; this is noted as a limitation in Section “4.1 Strengths and limitations.”

### 2.4 Variable definitions and outcome measures

OFDA usage frequency was dichotomized as: frequent users (daily, 4–6 times/week, or 2–3 times/week) and infrequent users (once/week or once/month), consistent with prior validated classifications in this field (20, 21). Body mass index (BMI) was computed from self-reported height and weight as weight(kg)/height(m)^2^ and categorized per World Health Organization standards: underweight (< 18.5 kg/m^2^), normal weight (18.5–24.9 kg/m^2^), overweight (25.0–29.9 kg/m^2^), Class I obesity (30.0–34.9 kg/m^2^), Class II obesity (35.0–39.9 kg/m^2^), and Class III obesity (≥ 40.0 kg/m^2^). Height values entered in meters (*n* = 5; range 1.0–1.75) were converted to centimeters prior to BMI calculation. Cuisine preferences were dichotomized for secondary analysis: healthy (vegetarian, salads, keto diet) versus unhealthy (fast food, desserts). This dichotomy drew on established nutrient-density and dietary-pattern frameworks rather than the menu composition of any single OFDA platform: fast food and desserts are consistently characterized in the nutrition literature as energy-dense and disproportionately high in saturated fat, refined sugar, and sodium (7–9, 22), whereas vegetarian dishes, salads, and ketogenic preparations are associated with higher vegetable content and lower energy density, in line with World Health Organization healthy-diet principles (22). Saudi, Arab, and international cuisines were not assigned to either category, as each spans a broad range of dishes with widely varying nutritional profiles that preclude a single valid classification; this simplification is acknowledged as a limitation in Section “4.1 Strengths and limitations.”

### 2.5 Statistical analysis

All analyses were conducted using IBM SPSS Statistics version 26.0 (IBM Corporation, Armonk, NY, USA). Categorical variables were summarized as frequencies and percentages. Before inferential testing, normality was assessed using the Shapiro–Wilk test and homogeneity of variance using Levene’s test; both assumptions were violated across Likert-scale items, warranting the exclusive use of nonparametric methods. Chi-square (χ^2^) tests of independence assessed associations between sociodemographic variables and OFDA usage frequency; where expected cell counts were < 5, Fisher’s exact test was applied. The Mann–Whitney *U* test compared Likert-scale perception scores between two independent groups (biological sex; frequent vs. infrequent users). The Kruskal–Wallis *H* test was used to evaluate differences across three or more independent groups (age category, region, employment status, income level). Pairwise *post-hoc* comparisons following significant Kruskal–Wallis results were conducted using Dunn’s test with Bonferroni correction. The association between OFDA frequency and dietary healthfulness was evaluated using a χ^2^ test; the relationship between OFDA frequency and BMI was assessed using the Mann–Whitney *U* test. All tests were two-tailed; statistical significance was set at *p* < 0.05.

## 3 Results

### 3.1 Sociodemographic characteristics and OFDA usage patterns

A total of 303 participants met eligibility criteria and were included in the analysis (Table 1). The sample was predominantly female (73.2%), Saudi nationals (94.4%), single (75.5%), and concentrated in the 20–29-year age group (70.5%). Most participants held a bachelor’s degree (67.5%) and resided with their family (90.4%). The Central Province was the most represented region (58.9%), followed by the Western Province (24.2%). Over half of participants (53.6%) reported monthly incomes below SAR 5,000, consistent with a large student and young-professional subsample.

Regarding OFDA usage frequency, 34.4% reported using delivery applications 2–3 times per week, 8.9% used them 4–6 times per week, and 8.3% reported daily use. Overall, 157 participants (51.7%) were classified as frequent users. Chi-square analysis identified significant associations between OFDA usage frequency and geographic region (χ^2^ = 10.48, df = 4, *p* = 0.033) and monthly income (χ^2^ = 9.53, df = 3, *p* = 0.023). Eastern Province residents demonstrated the highest rate of frequent use (70.4%), while Northern Province residents exhibited the lowest (22.2%). Among income groups, those earning SAR 5,001–10,000/month had the highest frequency (65.8%). No significant associations were observed between OFDA usage frequency and age (*p* = 0.166), biological sex (*p* = 0.165), nationality (*p* = 0.391), marital status (*p* = 0.111), educational attainment (*p* = 0.647), employment status (*p* = 0.064), or living situation (*p* = 0.056).

**TABLE 1:** Sociodemographic characteristics, OFDA usage frequency, and food choice determinants (*n* = 303).

Variable	Total *n* (%)	Frequent *n* (%)	Infrequent *n* (%)	*p*-value
Total	303 (100)	157 (51.7)	146 (48.3)	
Age (years)				
20–29	213 (70.5)	112 (52.6)	101 (47.4)	0.166
30–39	71 (23.5)	40 (56.3)	31 (43.7)	
≥ 40	19 (6.3)	5 (26.3)	14 (73.7)	
Biological sex				
Male	81 (26.8)	42 (51.9)	39 (48.1)	0.165
Female	221 (73.2)	115 (52.0)	106 (48.0)	
Nationality				
Saudi	285 (94.4)	146 (51.2)	139 (48.8)	0.391
Non-Saudi	18 (5.9)	11 (61.1)	7 (38.9)	
Marital status				
Single	228 (75.5)	124 (54.4)	104 (45.6)	0.111
Married	66 (21.9)	33 (50.0)	33 (50.0)	
Divorced	8 (2.6)	0 (0.0)	8 (100.0)	
Educational attainment				
High school	50 (16.6)	27 (54.0)	23 (46.0)	0.647
Diploma	17 (5.6)	9 (52.9)	8 (47.1)	
Bachelor	204 (67.5)	103 (50.5)	101 (49.5)	
Postgraduate	29 (9.6)	15 (51.7)	14 (48.3)	
Employment status				
Student	74 (24.5)	37 (50.0)	37 (50.0)	0.064
Government sector	57 (18.9)	27 (47.4)	30 (52.6)	
Private sector	105 (34.8)	63 (60.0)	42 (40.0)	
Self-employed	7 (2.3)	6 (85.7)	1 (14.3)	
Unemployed	59 (19.5)	24 (40.7)	35 (59.3)	
Region of Saudi Arabia				
Central Province	178 (58.9)	99 (55.6)	79 (44.4)	0.033*
Western Province	73 (24.2)	32 (43.8)	41 (56.2)	
Eastern Province	27 (8.9)	19 (70.4)	8 (29.6)	
Southern Province	15 (5.0)	5 (33.3)	10 (66.7)	
Northern Province	9 (3.0)	2 (22.2)	7 (77.8)	
Living situation				
With family	273 (90.4)	135 (49.5)	138 (50.5)	0.056
Alone	24 (7.9)	19 (79.2)	5 (20.8)	
With roommate	4 (1.3)	3 (75.0)	1 (25.0)	
Monthly income				
< SAR 5,000	162 (53.6)	71 (43.8)	91 (56.2)	0.023*
SAR 5,001–10,000	73 (24.2)	48 (65.8)	25 (34.2)	
SAR 10,001–15,000	50 (16.6)	29 (58.0)	21 (42.0)	
> SAR 15,000	17 (5.6)	9 (52.9)	8 (47.1)	
OFDA frequency of use				
Daily	25 (8.3)	—	—	
4–6 times/week	27 (8.9)	—	—	
2–3 times/week	104 (34.4)	—	—	
Once/week	74 (24.5)	—	—	
Once/month	72 (23.8)	—	—	
Food choice determinants (multiple response)				
Price	250 (82.8)	124 (49.6)	126 (50.4)	0.094
Food appearance	171 (56.6)	97 (56.7)	74 (43.3)	0.052
Delivery timing	150 (49.7)	83 (55.3)	67 (44.7)	0.274
Hygienic status of restaurant	130 (43.0)	69 (53.1)	61 (46.9)	0.703
Healthy options availability	89 (29.5)	49 (55.1)	40 (44.9)	0.467
Display nutrient/calorie content	73 (24.2)	31 (42.5)	42 (57.5)	0.067

*p*-values derived from Pearson chi-square test (*p < 0.05). OFDA, online food delivery application; SAR, Saudi Arabian Riyal.

### 3.2 Food choice determinants and application usage patterns

Price was the most frequently cited food-choice determinant (82.8%), followed by food appearance (56.6%), delivery timing (49.7%), restaurant hygienic status (43.0%), availability of healthy options (29.5%), and display of nutrient/calorie content (24.2%; Table 1). HungerStation was the most commonly used application (61.6%), followed by Jahez (53.3%), To You (37.4%), Keeta (27.2%), Ninja (21.5%), The Chefz (18.2%), and Marsool (14.6%). When participants were asked whether they actively sought healthy food options when ordering, 58.9% responded affirmatively. However, when asked about actual cuisine preferences, fast food was selected by 78.5% of participants, followed by desserts (41.1%), Saudi cuisine (33.8%), salads (31.1%), international cuisine (29.5%), Arab cuisine (21.5%), vegetarian food (7.6%), and keto diet items (4.3%).

### 3.3 Perceptions of healthy food on OFDAs

Table 2 presents mean scores ( ± SD), medians, and inferential test results for all seven healthy-food perception items (A1–A7). The statement receiving the highest mean was A1 (“I often find it challenging to find healthy food choices on food apps”; mean 3.78 ± 1.10), while A3 (“Using OFDAs has made me aware of healthier food alternatives”; mean 2.78 ± 1.21) received the lowest score, indicating that OFDAs are not widely perceived as helpful guides for healthy eating. More than half of the participants agreed or strongly agreed that OFDA use has changed their eating habits (A2; mean 3.48 ± 1.25) and increased their food intake and appetite (A7; mean 3.54 ± 1.22).

**TABLE 2:** Sociodemographic and behavioral predictors of healthy food perceptions among OFDA users (*n* = 303).

Item	Mean	SD	Median	Sex†*p*	Freq. use†*p*	Age‡ *p*	Employ.‡ *p*	Income‡ *p*
A1: Difficulty finding healthy options	3.78	1.10	4	0.115	0.024*	0.776	0.051	0.119
A2: OFDAs changed eating habits	3.48	1.25	4	0.450	< 0.001*	0.293	0.206	0.395
A3: OFDAs increase awareness of healthy alternatives	2.78	1.21	3	0.692	0.799	0.085	0.001*	0.094
A4: Macronutrient label affects food choice	3.46	1.27	4	0.056	0.288	0.234	0.367	0.014*
A5: Calorie label affects food choice	3.64	1.26	4	0.086	0.655	0.325	0.976	0.162
A6: Willing to pay more for healthy food	3.00	1.26	3	0.113	0.690	0.040*	0.937	0.743
A7: OFDAs increased appetite/food intake	3.54	1.22	4	0.577	0.020*	0.218	0.662	0.170

†Mann–Whitney *U* test (two independent groups).

‡Kruskal–Wallis *H* test (≥ 3 groups); *post-hoc* pairwise comparisons with Dunn–Bonferroni correction applied where *H*-test was significant.

*p < 0.05. Region *p*-values for A1–A7 were all non-significant (range 0.323–0.983) and are omitted for brevity but available in Supplementary Table 1. SD, standard deviation.

OFDA usage frequency was a significant independent predictor of perception scores for three items: A1—difficulty finding healthy options (*U* = 13,029.0, *p* = 0.024), A2—changed eating habits (*U* = 14,929.5, *p* < 0.001), and A7—increased appetite (*U* = 13,104.0, *p* = 0.020). Employment status significantly differentiated participants on A3—awareness of healthier alternatives (*H* = 19.13, *p* = 0.001; *post-hoc* analysis showed self-employed and government-sector employees had higher awareness than students and unemployed individuals) and B7—utility of in-app hygiene ratings (*H* = 15.22, *p* = 0.004). Income level significantly moderated the influence of macronutrient labeling on food choices (A4: *H* = 10.65, *p* = 0.014), with higher-income participants reporting greater susceptibility to nutritional information. Age was a significant predictor only of willingness to pay more for healthy food (A6: *H* = 8.32, *p* = 0.040), with younger participants (20–29 years) being less willing. Gender, living situation, age, and region showed no significant associations with items A1–A7 beyond those noted.

### 3.4 Perceptions of food safety and delivery hygiene

The overall mean food safety perception score across all seven B-items was 3.90/5.00 (Table 3), indicating moderately positive perceptions of food safety. The utility of restaurant hygiene ratings displayed within OFDAs (B7) achieved the highest agreement (mean 4.56 ± 0.72) of all 14 Likert items in the study, reflecting near-universal consensus. Meal packaging style (B5; mean 4.00 ± 0.97) and temperature as an indicator of food quality (B2; mean 3.93 ± 1.09) also received high scores, while the perception that items are prepared and delivered under sanitary conditions (B6; mean 3.62 ± 0.90) was more moderate, indicating residual uncertainty about delivery hygiene.

**TABLE 3:** Sociodemographic and behavioral predictors of food safety and delivery hygiene perceptions among OFDA users (*n* = 303).

Item	Mean	SD	Median	Sex†*p*	Freq. use†*p*	Age‡ *p*	Employ.‡ *p*	Income‡ *p*
B1: Meal temperature indicates safety	3.83	1.08	4	0.276	0.318	0.106	0.369	0.815
B2: Meal temperature indicates quality	3.93	1.09	4	0.365	0.467	0.307	0.780	0.765
B3: Driver appearance affects hygiene perception	3.84	1.03	4	0.001*	0.528	0.066	0.259	0.562
B4: Eco-friendly packaging influences food choice	3.52	1.18	4	0.025*	0.779	0.007*	0.300	0.733
B5: Packaging style influences food choice	4.00	0.97	4	0.019*	0.324	0.023*	0.101	0.509
B6: Items prepared/delivered under sanitary conditions	3.62	0.90	4	0.087	0.325	0.148	0.069	0.307
B7: In-app hygiene rating would be useful	4.56	0.72	5	0.418	0.623	0.348	0.004*	0.002*

†Mann–Whitney *U* test.

‡Kruskal–Wallis *H* test; *post-hoc* Dunn–Bonferroni correction applied for significant *H*-tests. Region *p*-values: B1: 0.009*; B7: 0.030*; all others non-significant (full data in Supplementary Table 1).

*p < 0.05. SD, standard deviation.

Biological sex was a significant moderator of perceptions regarding driver appearance (B3: *U* = 11,123.5, *p* = 0.001), eco-friendly packaging (B4: *U* = 10,411.0, *p* = 0.025), and packaging style (B5: *U* = 10,442.5, *p* = 0.019), with female participants consistently expressing stronger agreement. Region of residence significantly differentiated perceptions of meal temperature as a safety indicator (B1: *H* = 13.54, *p* = 0.009), with Central Province participants showing lower safety-from-temperature associations than residents of Eastern or Western Province. Employment status and income level significantly influenced perceived utility of in-app hygiene ratings (B7: employment *H* = 15.22, *p* = 0.004; income *H* = 14.44, *p* = 0.002), suggesting that employed and higher-income individuals place a greater priority on institutional food safety governance.

### 3.5 Association between BMI and OFDA usage frequency

BMI data were available and plausible for 302 of the 303 participants (mean 23.56 ± 5.23 kg/m^2^; Table 4). The largest proportion fell within the normal weight category (*n* = 154; 51.0%), followed by overweight (*n* = 71; 23.5%), underweight (*n* = 48; 15.9%), Class I obesity (*n* = 18; 6.0%), Class II obesity (*n* = 7; 2.3%), and Class III obesity (*n* = 4; 1.3%). A Mann–Whitney *U* test revealed no statistically significant difference in BMI between frequent (mean rank 150.27; *n* = 156) and infrequent users (mean rank 149.72; *n* = 146) (*U* = 11,723.0, *Z* = −0.44, *p* = 0.659), indicating that OFDA usage frequency was not independently associated with weight status in this sample.

**TABLE 4:** BMI distribution and association with OFDA usage frequency (*n* = 302).

BMI category (kg/m^2^)	Total *n* (%)	Frequent *n* (%)	Infrequent *n* (%)
Underweight (< 18.5)	48 (15.9)	26 (54.2)	22 (45.8)
Normal weight (18.5–24.9)	154 (51.0)	78 (50.6)	76 (49.4)
Overweight (25.0–29.9)	71 (23.5)	37 (52.1)	34 (47.9)
Class I obesity (30.0–34.9)	18 (6.0)	9 (50.0)	9 (50.0)
Class II obesity (35.0–39.9)	7 (2.3)	3 (42.9)	4 (57.1)
Class III obesity (≥ 40.0)	4 (1.3)	3 (75.0)	1 (25.0)
Mean BMI (± SD)	23.56 ± 5.23	23.69 ± 5.60	23.42 ± 4.81
Mann–Whitney *U*; *p*-value	–	11,723.0	0.659

Mann–Whitney *U* test comparing BMI between frequent and infrequent users. BMI, body mass index; SD, standard deviation.

### 3.6 OFDA usage frequency and healthfulness of food choices

Among participants who selected exclusively healthy (vegetarian, salads, keto diet) or exclusively unhealthy (fast food, desserts) cuisine types and were therefore classifiable (*n* = 194), the distribution of food choices did not differ significantly by OFDA usage frequency (χ^2^ = 0.00, df = 1, *p* = 1.000; Table 5). Unhealthy selections predominated across frequency groups: 76 of 88 classifiable frequent users (86.4%) and 91 of 106 classifiable infrequent users (85.8%) selected unhealthy options, while only 12 (13.6%) and 15 (14.2%) selected healthy options, respectively.

**TABLE 5:** Association between OFDA usage frequency and dietary healthfulness of food choices (*n* = 194).

Food choice category	Frequent *n* (%)	Infrequent *n* (%)	χ ^2^ (df = 1); *p*-value
Healthy (vegetarian/salads/keto diet)	12 (13.6)	15 (14.2)	0.00; *p* = 1.000
Unhealthy (fast food/desserts)	76 (86.4)	91 (85.8)	
Total classifiable participants	88	106	

Participants selecting Saudi, Arab, or international cuisines exclusively were excluded from this analysis (*n* = 109) due to heterogeneous nutritional profiles. *p*-value from Pearson chi-square test.

## 4 Discussion

This study examined the frequency of OFDA use and its sociodemographic determinants, the primary factors influencing food choices, and user perceptions of healthy food availability and food safety among 303 Saudi residents aged 20 years and older. The main findings were: (i) 51.7% of participants were classified as frequent OFDA users; (ii) price (82.8%) was the dominant food-choice determinant, followed by food appearance (56.6%) and delivery timing (49.7%); (iii) despite 58.9% of participants reporting they seek healthy options, fast food was the most ordered cuisine (78.5%); (iv) frequent OFDA use was significantly associated with changed eating habits, increased appetite, and difficulty locating healthy options; (v) food safety perceptions were moderately positive, with near-universal agreement on the utility of in-app hygiene ratings; and (vi) OFDA usage frequency was not associated with BMI or dietary healthfulness.

Over half of participants (51.7%) were classified as frequent OFDA users in the present sample, with the largest proportion (34.4%) using these applications 2–3 times per week. This prevalence is comparable to the 52.0% frequent-use rate reported by Cheikh Ismail et al. (20) among UAE university students. The predominance of participants aged 20–29 years (70.5%) among frequent users is consistent with findings from Saudi Arabia and the wider MENA region, where younger adults demonstrate higher rates of OFDA engagement (5, 23, 24). HungerStation was the most-used application (61.6%), followed by Jahez (53.3%), consistent with the ranking reported by Alnasser and Abaalkhail (5), who identified HungerStation as the leading platform in Saudi Arabia (55.3%), though the higher rates in our study may reflect continued market growth since that data collection period. Region of residence and monthly income were the only sociodemographic variables significantly associated with usage frequency (*p* = 0.033 and *p* = 0.023, respectively), in line with evidence that geographic access to restaurants and disposable income are key structural determinants of takeout food consumption frequency (21). A plausible explanation for the regional disparity is that Eastern Province cities such as Dammam and Al-Khobar combine higher average household income from the petroleum sector with denser OFDA delivery infrastructure and shorter average delivery distances, both of which lower the practical and financial barriers to frequent ordering, whereas the predominantly rural Northern Province may have more limited restaurant density and delivery coverage (4, 5).

Price emerged as the most influential factor affecting food choices in this sample (82.8%), followed by food appearance (56.6%) and delivery timing (49.7%). These findings are in agreement with comparable studies in the region. Among UAE university students, Cheikh Ismail et al. (20) similarly found that price (78.0%) and food appearance (65.7%) were the two leading food-choice factors. Saleh et al. (10) reported that price and appearance together accounted for 65.0% of food selection decisions among adolescent OFDA users in the UAE, and a study in Kuwait found that price was also a primary determinant of food ordering behavior (11). Notably, less than one quarter of participants in the present study (24.2%) cited nutrient or calorie content as a food-choice factor, indicating that nutritional labeling currently has limited influence on ordering decisions in Saudi Arabia. Income level significantly moderated the effect of macronutrient labeling on food choices (A4: *p* = 0.014), suggesting that higher-income participants are more responsive to nutritional information. This finding is consistent with the literature, which shows that lower-income consumers tend to prioritize cost over nutritional content, while higher-income individuals are more likely to consider nutritional value when making food purchases (25–27). A plausible explanation for this income gradient is the cognitive and financial trade-off inherent in nutrition label use: processing and acting on displayed nutrient information requires sufficient discretionary income to select a higher-priced, better-labeled item, whereas price-constrained consumers must prioritize the most economical option regardless of its nutritional content, a pattern previously documented among nutrition label users in other consumer settings (27).

A notable finding in the present study was the discrepancy between stated health intentions and actual food choices. Although 58.9% of participants reported actively seeking healthy food options when ordering through OFDAs, fast food was the most frequently ordered cuisine (78.5%), followed by desserts (41.1%). This intention-behavior gap has been consistently reported in similar OFDA studies. Among UAE university students, Cheikh Ismail et al. (20) found that 69.3% reported seeking healthy food options, yet unhealthy foods remained the most popular overall. Eu and Sameeha (12) observed that while 85.9% of Malaysian university students expressed interest in ordering healthy food online, 77.6% ultimately selected unhealthy items, with limited availability and higher cost identified as the main barriers. A similar pattern was reported in Saudi Arabia and Kuwait (11, 23). The statement “Using OFDAs has made me aware of healthier food alternatives” (A3) received the lowest mean ± SD among the 14 perception items (2.78 ± 1.21), indicating that current OFDA platforms do not effectively guide users toward nutritious choices. The dominance of fast food on OFDA menus—where energy-dense items tend to appear in more prominent positions and receive greater promotional marketing—is likely a contributing factor to this pattern (26, 28). From a choice-architecture perspective, this pattern is consistent with evidence that altering the relative visibility and default positioning of options within a decision environment can substantially shape behavior even when the full range of choices remains technically available (29); because OFDA interfaces during the study period algorithmically prioritized fast-food and dessert listings over healthier categories, users seeking nutritious alternatives likely encountered a structurally disadvantaged search process that favored impulsive, default selections over deliberate healthy choices.

Frequent OFDA users in this sample were significantly more likely than infrequent users to report that using these applications had changed their eating habits (A2: *p* < 0.001), increased their appetite and food intake (A7: *p* = 0.020), and that they found it challenging to locate healthy options (A1: *p* = 0.024). These findings are consistent with Cheikh Ismail et al. (20), who reported that frequent OFDA users among UAE university students had significantly lower healthy food perception scores, and that 57.8% of all users agreed that OFDA use had increased their appetite and food intake, a proportion comparable to the mean agreement level observed for A7 in our sample. Similar associations between OFDA use frequency and dietary behavior change have been reported in Kuwait (11) and Brazil (28). The significant effect of frequency on perceived difficulty finding healthy options (A1) may reflect greater familiarity with OFDA menus among frequent users, making the limited availability of nutritious choices more apparent over time. Employment status was also an independent predictor of awareness of healthier food alternatives through OFDAs (A3: *p* = 0.001), with *post hoc* analysis indicating higher awareness among self-employed and government-sector workers than among students and unemployed individuals. This may be attributed to differences in nutrition literacy associated with occupational context, as adults in formal employment may have greater access to occupational health programs or health-promotion resources (25). Workplace-based wellness initiatives and the generally higher health literacy associated with stable formal employment may equip self-employed and government-sector workers with a greater capacity to recognize and actively seek out the limited healthy options embedded within OFDA menus, whereas students and unemployed individuals, who reported comparatively lower awareness in the present study, may have had less structured exposure to such health-promoting information.

Participants in this sample expressed moderately positive food safety perceptions, with an overall mean score of 3.90 out of 5.00. The perception that in-app restaurant hygiene ratings would be useful (B7) achieved the highest mean score of all 14 items in the study (4.56 ± 0.72), reflecting near-universal consensus. This finding corroborates Cheikh Ismail et al. (20), who reported that 82.3% of UAE university students agreed that an in-app hygiene rating system would help them make more informed ordering decisions, and Saleh et al. (10), who similarly found strong support for hygiene transparency among UAE adolescent OFDA users. Meal temperature was regarded as an important indicator of both food safety (B1: mean 3.83 ± 1.08) and food quality (B2: mean 3.93 ± 1.09), consistent with evidence that temperature maintenance during delivery is a key determinant of consumer food safety perceptions (13, 14). Female participants in this sample expressed significantly stronger agreement with several food safety items, including the effect of driver appearance on hygiene perception (B3: *p* = 0.001), eco-friendly packaging (B4: *p* = 0.025), and meal packaging (B5: *p* = 0.019). This is consistent with Saleh et al. (10), who reported that female adolescent OFDA users had significantly higher food safety perception scores than their male counterparts, and with broader evidence showing that women tend to be more sensitive to food hygiene and packaging practices (10, 30). This gender difference may be attributable to women’s traditionally greater involvement in household food provisioning and meal preparation in the Saudi context, which could heighten sensitivity to visual and contextual cues signaling food safety and quality even when ordering through a third-party delivery platform rather than preparing the meal directly. Employment status and income were both significant predictors of perceived utility of hygiene ratings (B7: *p* = 0.004 and *p* = 0.002, respectively), suggesting that employed and higher-income individuals place greater value on formal food safety governance.

No statistically significant difference in BMI was observed between frequent and infrequent OFDA users in this sample (*U* = 11,723.0, *p* = 0.659), and OFDA usage frequency was not associated with the healthfulness of food choices [χ^2^(1) = 0.00, *p* = 1.000]. These null findings are consistent with Keeble et al. (21), who, across a multi-country analysis, reported that OFDA use was not associated with weight status, noting that the diverse menu offerings on these platforms—including some nutritious options—may dilute any direct relationship with BMI. Sony et al. (31) similarly found no significant association between use of food delivery applications and BMI among students, despite observations of higher fat intake among frequent users. The absence of a BMI association here may be partly explained by the cross-sectional design, which captures a single time point and cannot account for cumulative dietary exposure. Self-reported height and weight are also subject to reporting bias, which may have attenuated a true association. It is also plausible that any weight-related impact of habitual OFDA use requires a longer latency period to manifest as a measurable change in BMI than a single cross-sectional assessment can capture, particularly if frequent users behaviorally compensate, for example, by adjusting portion sizes, skipping subsequent meals, or increasing physical activity in ways not captured by the present study’s measures. The parallel null finding for dietary healthfulness indicates that usage frequency alone is not a useful predictor of dietary quality; rather, food choices appear to be driven primarily by price, availability, and individual characteristics, independent of how often one uses OFDAs.

The regulatory timeline described in the Introduction is directly relevant to interpreting the present findings. Because mandatory nutritional labeling was only beginning to be enforced during the survey period, the limited reported influence of nutrient and calorie labeling on food choice (Section “3.2 Food choice determinants and application usage patterns”) may partly reflect the near-absence of such labeling on Saudi OFDA platforms at the time of data collection, rather than a genuine lack of consumer interest in nutritional information. Conversely, the near-universal support observed for in-app hygiene ratings (Section “3.4 Perceptions of food safety and delivery hygiene”) anticipated and is consistent with the direction of the delivery-hygiene reforms subsequently introduced under the national Food System Violations Table. Taken as a whole, these findings offer empirical, population-level evidence to inform the ongoing implementation of emerging Saudi food regulations, particularly by identifying which sociodemographic subgroups may require targeted outreach to ensure the new labeling and hygiene requirements translate into meaningful behavior change rather than remain underused.

Taken together, these findings argue for a more developed set of regulatory and platform-design interventions than has typically been implemented to date, extending beyond the brief summary offered in the Conclusion. Mandatory nutritional labeling, already being phased in by the SFDA for physical and digital menus (15), could be operationalized within OFDA interfaces as standardized front-of-listing nutrient badges or traffic-light color coding rather than text-based disclosures alone, which are more easily overlooked during rapid in-app browsing. However, mandating labeling alone is unlikely to be sufficient: a benchmarking analysis of OFDA platforms in Sydney, Australia, found that despite an existing legal requirement, kilojoule-labeling compliance varied widely across platforms, from 22.5% to 88% of menu items depending on the specific application (32), indicating that compliance monitoring and enforcement mechanisms must accompany any labeling mandate introduced in Saudi Arabia. Algorithmic promotion of healthy options represents a complementary, lower-cost intervention: consistent with the choice-architecture framework discussed above (29), platforms could default to a “balanced” or “healthy” sort order, increase the visual prominence of a dedicated healthy-options filter, or limit the algorithmic preferencing currently given to fast-food and dessert listings in search results and promotional banners. In-app hygiene rating integration has a workable precedent in the United Kingdom, where the Food Standards Agency negotiated a Food Safety Charter with three major delivery platforms requiring verification of restaurant registration, in-app display of official hygiene ratings, and removal of zero-rated restaurants from listings (33); given that Saudi Arabia’s own Food System Violations Table already formalizes delivery-specific hygiene oversight (16), extending similar in-app display requirements to Saudi OFDA platforms would represent a natural and immediately actionable next step. Finally, price-equity mechanisms, such as waived or reduced delivery fees for nutritionally superior menu items, subsidized healthy meal bundles, or tax incentives for restaurants and platforms that expand healthy offerings, would directly address the price-primacy barrier identified as the dominant food-choice determinant in the present sample (Section “3.2 Food choice determinants and application usage patterns”), and may be necessary if labeling and algorithmic interventions alone are to translate into meaningfully different consumer behavior rather than simply better-informed but unchanged choices.

### 4.1 Strengths and limitations

The present study has several strengths. It is among the first to examine OFDA usage patterns, food-choice determinants, and food safety perceptions simultaneously in a multi-regional Saudi adult sample spanning all five geographic regions. The questionnaire was adapted from instruments previously validated in comparable MENA populations (11, 18, 20), appropriate nonparametric statistical methods were applied given violations of distributional assumptions, and the final sample (*n* = 303) exceeded the prespecified minimum of 300. Several limitations should, however, be considered. First, the cross-sectional design does not allow causal inference; the observed associations cannot be interpreted as evidence of causality. Second, convenience and snowball sampling may have introduced selection bias; the predominance of young, female, educated, and Central Province participants limits the generalizability of the findings to older, male, rural, and less educated Saudi populations. Third, self-reported height and weight are prone to social desirability bias and may have led to underestimation of overweight and obesity prevalence. Fourth, social desirability may have inflated the proportion of participants reporting health-seeking intentions. Fifth, the binary classification of food choices does not capture the nutritional diversity of Saudi, Arab, and international cuisines, which were excluded from this analysis due to their heterogeneous nutritional profiles. Sixth, although the adapted Likert subscales demonstrated acceptable internal consistency when assessed *post hoc* in the present sample (Section “2.3 Survey instrument”), the instrument had not undergone separate psychometric validation (e.g., confirmatory factor analysis, test–retest reliability) prior to fielding in this population. Future studies should employ longitudinal designs, validated dietary assessment methods such as 24-h dietary recalls or food frequency questionnaires, and objective anthropometric measurements to provide a more accurate characterization of the long-term dietary consequences of OFDA use in the Saudi population.

## 5 Conclusion

Online food delivery applications are deeply embedded in the dietary lives of young Saudi adults, primarily facilitating the purchase of fast food and energy-dense snacks driven by price sensitivity and convenience. The prominent intention–behavior gap—between health-seeking aspirations and actual food ordering—reflects the structural dominance of unhealthy options within current OFDA architectures rather than a simple absence of health motivation. Frequent users report greater difficulty finding healthy options, greater dietary habit change, and heightened appetite than infrequent users, yet frequency of use does not independently predict BMI or dietary healthfulness. Food safety perceptions are moderately positive, with near-universal support for mandatory in-app displays of hygiene ratings. These findings argue for multi-pronged regulatory and platform-design interventions: mandatory nutritional labeling, algorithmic promotion of healthy options, integration of in-app hygiene ratings, and price-equity mechanisms to reduce the cost differential between nutritious and energy-dense meals. Longitudinal research tracking dietary quality and anthropometric outcomes over sustained OFDA use remains a critical priority for the field.

## Data Availability

The data supporting the findings of this study are available from the corresponding author upon reasonable request.

## References

[1] Harari GM Lane ND Wang R Crosier BS Campbell AT Gosling SD. Using smartphones to collect behavioral data in psychological science: opportunities. *Perspect Psychol Sci*. (2016) 11:838–54. 10.1177/1745691616650285 27899727 PMC5572675

[2] Bennett R Keeble M Zorbas C Sacks G Driessen C Grigsby-Duffy L et al. The potential influence of the digital food retail environment on health: a systematic scoping review of the literature. *Obes Rev*. (2024) 25:e13671. 10.1111/obr.13671 38104965

[3] Zaheer MA Anwar TM Iantovics LB Raza MA Khan Z. Enticing attributes of consumers’ purchase intention to use online food delivery applications (OFDAs) in a developing country. *J Bus Econ.* (2023) 3:1–18. 10.3934/jbe.2023010

[4] Asfoura E Aljuraywi N Alhajjaj A Alzahed A. Investigating the role of digital emerging enterprises specialized in food delivery service in Saudi context. *Int J Manag.* (2021) 12:1043–56. 10.34218/IJM.12.3.2021.085

[5] Alnasser A Abaalkhail A. Digital food behaviours, motivations, and delivery application usage among saudis during COVID-19: a mixed-methods study. *Heliyon*. (2024) 10:e24903. 10.1016/j.heliyon.2024.e24903 38317926 PMC10840008

[6] Alhusseini N Alqahtani A. COVID-. 19 pandemic’s impact on eating habits in Saudi Arabia. *J Public Health Res*. (2020) 9:1868. 10.4081/jphr.2020.1868 33024727 PMC7512943

[7] Lachat C Nago E Verstraeten R Roberfroid D Van Camp J Kolsteren P. Eating out of home and its association with dietary intake: a systematic review of the evidence. *Obes Rev*. (2012) 13:329–46. 10.1111/j.1467-789X.2011.00953.x 22106948

[8] Bezerra IN Curioni C Sichieri R. Association between eating out of home and body weight. *Nutr Rev*. (2012) 70:65–79. 10.1111/j.1753-4887.2011.00459.x 22300594

[9] Mahawar N Jia SS Korai A Wang C Allman-Farinelli M Chan V et al. Unhealthy Food at Your Fingertips: Cross-Sectional Analysis of the Nutritional Quality of Restaurants and Takeaway Outlets on an Online Food Delivery Platform in New Zealand. *Nutrients*. (2022) 14:4567. 10.3390/nu14214567 36364829 PMC9656530

[10] Saleh ST Osaili TM Al-Jawaldeh A Hasan HA Hashim M Mohamad MN et al. Adolescents’ use of online food delivery applications and perceptions of healthy food options and food safety: a cross-sectional study in the United Arab Emirates. *Front Nutr*. (2024) 11:1385554. 10.3389/fnut.2024.1385554 38628272 PMC11018892

[11] Almansour FD Allafi AR Zafar TA Al-Haifi AR. Consumer prevalence, attitude and dietary behavior of online food delivery applications users in Kuwait. *Acta Biomed*. (2020) 91:e2020178. 10.23750/abm.v91i4.8543 33525272 PMC7927546

[12] Eu EZR Sameeha MJ. Consumers’ perceptions of healthy food availability in online food delivery applications (OFD APPS) and its association with food choices among public university students in Malaysia. *Front Nutr* (2021) 8:674427. 10.3389/fnut.2021.674427 34497818 PMC8419248

[13] Christian M Wibowo S Yulita H Melati R Sunarno S Perdini FT. Two phases of online food delivery app users’ behavior in Greater Jakarta during the second year of the COVID-19 pandemic: perceptions of food safety and hygiene. *Environ Health Eng Manag J.* (2023) 10:249–59. 10.34172/EHEM.2023.27

[14] Al Amin M Arefin MS Sultana N Islam MR Jahan I Akhtar A. Evaluating the customers’ dining attitudes, e-satisfaction, and continuance intention toward mobile food ordering apps: evidence from Bangladesh. *Eur J Manag Bus Econ* (2020) 30:211–29. 10.1108/EJMBE-06-2020-0158

[15] Saudi Food and Drug Authority. *SFDA to Implement New Food Rules for Transparency and Consumer Health. Riyadh: SFDA.* (2025). Available online at: https://www.sfda.gov.sa/en/news/3745904 (accessed February 2026).

[16] Saudi Press Agency. *Ministry of Municipalities and Housing, SFDA Launch Updated Food System Violations Table. Riyadh: SPA.* (2025). Available online at: https://spa.gov.sa/en/N2387586 (accessed February 2026).

[17] Jamjoom AB Gahtani AY Sharab BM. Regional variation in the neurosurgical workforce in Saudi Arabia. *Cureus*. (2022) 14:e28236. 10.7759/cureus.28236 36158333 PMC9488865

[18] Cheikh Ismail L Osaili TM Mohamad MN Hashim M Stojanovska L Al Daour R et al. Psychosocial factors affecting dietary habits of university students: a cross-sectional study. *Heliyon*. (2022) 8:e09768. 10.1016/j.heliyon.2022.e09768 35789869 PMC9249847

[19] World Health Organization. *WHO STEPS Surveillance Manual: The WHO STEPwise Approach to Noncommunicable Disease Risk Factor Surveillance.* Geneva: WHO. (2017).

[20] Cheikh Ismail L Osaili TM Shanan B Rashwan D Merie H Rishan L et al. A cross-sectional study on online food delivery applications (OFDAs) in the United Arab Emirates: use and perceptions of healthy food availability among university students. *J Nutr Sci*. (2024) 13:e62. 10.1017/jns.2024.21 39464403 PMC11503857

[21] Keeble M Adams J Sacks G Vanderlee L White CM Hammond D et al. Use of online food delivery services to order food prepared away-from-home and associated sociodemographic characteristics: a cross-sectional. *Int J Environ Res Public Health*. (2020) 17:5190. 10.3390/ijerph17145190 32709148 PMC7400536

[22] de Ridder D Kroese F Evers C Adriaanse M Gillebaart M. Healthy diet: health impact, prevalence, correlates, and interventions. *Psychol Health*. (2017) 32:907–41. 10.1080/08870446.2017.1316849 28447854

[23] Alwafi H Fakeerh M Naser AY Khogeer AA Alotaibi FF Alkhamis AA. The impact of food delivery applications on food consumption: a cross-sectional online survey in Saudi Arabia. *Bahrain Med Bull.* (2024) 46:2070–4. 10.2139/ssrn.4518205 40556929

[24] Buettner SA Pasch KE Poulos NS. Factors associated with food delivery app use among young adults. *J Community Health*. (2023) 48:840–6. 10.1007/s10900-023-01229-1 37148460 PMC10163566

[25] Desbouys L Méjean C De Henauw S Castetbon K. Socio-economic and cultural disparities in diet among adolescents and young adults: a systematic review. *Public Health Nutr*. (2020) 23:843–60. 10.1017/S1368980019002362 31466544 PMC10200431

[26] Jia SS Todd AR Vanderlee L Farrell P Allman-Farinelli M Sacks G et al. Offline to online: a systematic mapping review of evidence to inform nutrition-related policies applicable to online food delivery platforms. *BMC Med*. (2024) 22:542. 10.1186/s12916-024-03747-8 39558372 PMC11575118

[27] Drichoutis AC Lazaridis P Nayga RM. Nutrition knowledge and consumer use of nutritional food labels. *Eur Rev Agric Econ.* (2005) 32:93–118. 10.1093/erae/32.1.93

[28] Horta PM Souza JPM Rocha LL Mendes LL. Digital food environment of a Brazilian metropolis: food availability and marketing strategies used by delivery apps. *Public Health Nutr*. (2021) 24:544–8. 10.1017/S1368980020003171 32900419 PMC10195591

[29] Hollands GJ Shemilt I Marteau TM Jebb SA Kelly MP Nakamura R et al. Altering micro-environments to change population health behaviour: towards an evidence base for choice architecture interventions. *BMC Public Health*. (2013) 13:1218. 10.1186/1471-2458-13-1218 24359583 PMC3881502

[30] Rokka J Uusitalo L. Preference for green packaging in consumer product choices – do consumers care? *Int J Consum Stud.* (2008) 32:516–25. 10.1111/j.1470-6431.2008.00710.x

[31] Sony AGC Ilmi IMB Sofianita N Octaria YC. Relationship between the use of food delivery applications, fat intake, physical activity, and weight status among students. *J Gizi Pangan.* (2024) 19:191–7. 10.25182/jgp.2024.19.Supp2.191-197

[32] Cassano S Jia A Gibson AA Partridge SR Chan V Farrell P et al. Benchmarking online food delivery applications against menu labelling laws: a cross-sectional observational analysis. *Public Health Nutr*. (2024) 27:e101. 10.1017/S1368980024000673 38557393 PMC11036439

[33] Food Standards Agency. *UK food delivery platforms agree new Food Safety Charter.* London: FSA. (2022). Available online at: https://www.food-safety.com/articles/8076-uk-takes-steps-to-regulate-online-food-sales-delivery-services (accessed February 2026).

