# Supplementary material for: Food choice determinants and perceptions of healthy eating and food safety among online food delivery application users in Saudi Arabia: a cross-sectional study
Source: Front Nutr. 2026 Aug 27;13:1887881. doi: 10.3389/fnut.2026.1887881 (PMC13558006; doi:10.3389/fnut.2026.1887881)
Supplement: Supplementary file 1 [file Table_1.docx]

**Supplementary Material S1: Survey Questionnaire**

*Manuscript Title: Food Choice Determinants and Perceptions of Healthy Eating and Food Safety Among Online Food Delivery Application Users in Saudi Arabia: A Cross-Sectional Study*

*Authors: Nawaf W. Alruwaili^1*^ (ORCID: 0000-0002-3375-8796; nalruwaili@ksu.edu.sa) | Abdulhmed Alharbi^1,2^ (ORCID: 0009-0008-9811-2698; 445108048@student.ksu.edu.sa)*

*1 Department of Community Health Sciences, College of Applied Medical Sciences, King Saud University, Riyadh 11433, Saudi Arabia*

*2 Clinical Nutrition Department, King Abdullah Specialized Hospital, Ministry of National Guard Health Affairs, Qassim, Saudi Arabia*

*Ethics Approval: King Saud University Standing Committee on Research Ethics (Ref: KSU-HE-25-094; approval date 11 February 2025)*

Instructions: By completing this survey, you agree to participate in this research study. Your responses are anonymous and confidential. There are no right or wrong answers; please respond based on your own experience and opinion.

**SECTION 1: Sociodemographic Information**

**Q1. Your completion of this survey indicates your consent to participate in the study.**

o Yes	o No

**Q2. What is your biological sex?**

o Male	o Female

**Q3. What is your nationality?**

o Saudi	o Non-Saudi

**Q4. What is your current marital status?**

o Single	o Married	o Divorced	o Widowed

**Q5. What is your age group?**

o 20-29 years	o 30-39 years	o 40-49 years	o 50-59 years	o 60 years or older

**Q6. What is your height? (Please enter numerically in centimeters)**

__________ cm

**Q7. What is your weight? (Please enter numerically in kilograms)**

__________ kg

**Q8. What is the highest level of education you have completed?**

o Middle school (Intermediate)	o High school	o Diploma	o Bachelor's degree	o Postgraduate (Master's/PhD)

**Q9. What is your current employment status?**

o Student	o Government sector employee	o Private sector employee	o Self-employed	o Unemployed/seeking employment	o Retired

**Q10. In which region of Saudi Arabia do you currently reside?**

o Central Province (Riyadh)	o Western Province (Makkah/Madinah)	o Eastern Province	o Southern Province	o Northern Province

**Q11. Who do you currently live with?**

o With family	o Alone	o With roommate(s)/colleagues

**Q12. What is your approximate monthly income/allowance?**

o Less than SAR 5,000	o SAR 5,001-10,000	o SAR 10,001-15,000	o More than SAR 15,000

**SECTION 2: Online Food Delivery Application Usage**

**Q13. How often do you use online food delivery applications?**

o Daily	o 4-6 times/week	o 2-3 times/week	o Once/week	o Once/month	o Never

*(Note: If you selected 'Never', the survey ends here. Thank you for your participation.)*

**Q14. Which online food delivery application(s) do you usually use? (Select all that apply)**

o HungerStation (هنقرستيشن)	o Jahez (جاهز)	o Marsool (مرسول)	o To You (تو يو)	o Cari (كاري)	o Ninja (نينجا)	o Mr. Mandoob (مستر مندوب)	o The Chefz (ذا شفز)	o Keeta (كيتا)	o Other (please specify below)

If 'Other', please specify: _______________

**Q15. What types of cuisine do you mostly look for when using food delivery applications? (Select all that apply)**

o Vegetarian/plant-based food	o Fast food	o International cuisine	o Saudi cuisine	o Arab cuisine	o Salads	o Keto diet	o Desserts

**Q16. Which of the following factors influence your food choices when using online food delivery applications? (Select all that apply)**

o Food appearance (color, texture, crunchiness)	o Price of the meal	o Delivery time	o Availability of healthy options	o Display of nutritional information and calorie content	o Hygienic rating/status of the restaurant

**Q17. When ordering through an online food delivery application, do you look for healthy food options?**

o Yes	o No

**SECTION 3: Perceptions of Healthy Food on Online Food Delivery Applications**

*Instructions: Please indicate your level of agreement with the following statements using the scale below:*

1 = Strongly disagree	2 = Disagree	3 = Neutral	4 = Agree	5 = Strongly agree

| **Statement** | **1** | **2** | **3** | **4** | **5** |
| --- | --- | --- | --- | --- | --- |
| A1. I often find it challenging to find healthy food choices on food delivery apps. | o | o | o | o | o |
| A2. Using online food delivery applications has changed my eating habits. | o | o | o | o | o |
| A3. Using online food delivery applications has made me aware of healthier food alternatives. | o | o | o | o | o |
| A4. My food choice is affected by the presence of macronutrient content labels on food items (e.g., fat, carbohydrate, and protein content). | o | o | o | o | o |
| A5. My food choice is affected by the presence of a calorie content label on food items. | o | o | o | o | o |
| A6. I am willing to pay a higher price for healthier food options. | o | o | o | o | o |
| A7. I feel that ordering food through online delivery applications has increased my appetite and overall food intake. | o | o | o | o | o |

**SECTION 4: Perceptions of Food Safety and Delivery Hygiene**

*Instructions: Please indicate your level of agreement with the following statements.*

1 = Strongly disagree	2 = Disagree	3 = Neutral	4 = Agree	5 = Strongly agree

| **Statement** | **1** | **2** | **3** | **4** | **5** |
| --- | --- | --- | --- | --- | --- |
| B1. The temperature of the delivered meal mainly gives me an impression of the safety of the food. | o | o | o | o | o |
| B2. The temperature of the delivered meal mainly gives me an impression of the quality of the food. | o | o | o | o | o |
| B3. The appearance of the delivery driver (cleanliness, neatness) affects my perception of the meal's hygiene. | o | o | o | o | o |
| B4. Having the meal delivered in environmentally friendly packaging materials influences my food choice. | o | o | o | o | o |
| B5. The packaging style of the meal influences my food choices (e.g., attractive packaging, colorful packaging, or securely sealed packaging). | o | o | o | o | o |
| B6. The food items available on delivery applications are prepared and delivered under hygienic conditions. | o | o | o | o | o |
| B7. It would be useful if restaurant or delivery applications displayed the hygiene rating of the restaurant when ordering. | o | o | o | o | o |

*--- END OF QUESTIONNAIRE ---*

**Supplementary Figure S1: Participant Recruitment Flow Diagram**
