# Supplementary material for: Food choice determinants and perceptions of healthy eating and food safety among online food delivery application users in Saudi Arabia: a cross-sectional study
Source: Front Nutr. 2026 Aug 27;13:1887881. doi: 10.3389/fnut.2026.1887881 (PMC13558006; doi:10.3389/fnut.2026.1887881)
Supplement: Supplementary file 2 [file Table_2.docx]

**STROBE Statement — Checklist of Items for Reports of Cross-Sectional Studies**

*Journal: Frontiers in Nutrition | Section: Nutritional Epidemiology*

*Manuscript: Food Choice Determinants and Perceptions of Healthy Eating and Food Safety Among Online Food Delivery Application Users in Saudi Arabia: A Cross-Sectional Study*

*Authors: Nawaf W. Alruwaili (ORCID: 0000-0002-3375-8796), Abdulhmed Alharbi (ORCID: 0009-0008-9811-2698)*

STROBE Reference: von Elm E, Altman DG, Egger M, Pocock SJ, Gotzsche PC, Vandenbroucke JP, et al. The Strengthening the Reporting of Observational Studies in Epidemiology (STROBE) statement: guidelines for reporting observational studies. Lancet. (2007) 370:1453-57.

| **Item** | **Section/Topic** | **Recommendation** | **Location in Manuscript** |
| --- | --- | --- | --- |
| 1a | **Title and abstract** | Indicate the study design with a commonly used term in the title or abstract | Title: '...A Cross-Sectional Study' stated explicitly. Abstract: structured single paragraph (Background, Objectives, Methods, Results, Conclusion) on p.1. |
| 1b |  | Provide an informative and balanced summary of what was done and what was found | Abstract (p.1): covers rationale, aims, methods, key results, and conclusion. |
| 2 | **Introduction: Background/rationale** | Explain the scientific background and rationale for the investigation | Introduction paragraphs 1-4 (pp.2-3): global OFDA growth, Saudi Arabia context, dietary quality evidence, food safety gaps. |
| 3 | **Introduction: Objectives** | State specific objectives, including any prespecified hypotheses | Introduction paragraph 5 (p.3): five explicit objectives stated. |
| 4 | **Methods: Study design** | Present key elements of study design early in the paper | Methods 2.1 (p.3): 'A cross-sectional, online observational study.' |
| 5 | **Methods: Setting** | Describe the setting, locations, and relevant dates | Methods 2.1-2.2 (p.3): Saudi Arabia; data collection February to April 2025; social media distribution. |
| 6a | **Methods: Participants** | Give eligibility criteria, and sources and methods of selection | Methods 2.2 (p.3-4): aged >=20 y, residing in Saudi Arabia, OFDA use >=once/month; excluded never-users. Convenience + snowball sampling via (Facebook, X [formerly Twitter], Instagram, and LinkedIn), disseminated organically via unpaid posts (no paid advertisements). The past-month usage threshold is explicitly justified as adopted from the identical criterion used by Saleh et al. (ref. 9). |
| 6b |  | For cross-sectional study: describe how study size was arrived at | Methods 2.2 (p.4): WHO formula (CI=95%, SD=0.5, margin of error=5%) -> minimum n=300; final n=303 (exceeded). |
| 7 | **Methods: Variables** | Clearly define all outcomes, exposures, predictors, potential confounders | Methods 2.3-2.4 (p.4-5): primary exposure = OFDA frequency (dichotomized); outcomes = Likert perception scores (A1-A7, B1-B7), BMI, dietary healthfulness; all variables defined. The healthy/unhealthy cuisine classification is now explicitly grounded in nutrient-density and WHO healthy-diet criteria, with rationale provided for excluding mixed-nutrient cuisine categories. |
| 8* | **Methods: Data sources/measurement** | Give sources of data and methods of assessment for each variable | Methods 2.3 (p.4): validated self-administered online questionnaire adapted from Osaili et al. (2024), Cheikh Ismail et al. (2024), and Almansour et al. (2020) (refs 17, 34, and 10). Five-point Likert scale (1=strongly disagree to 5=strongly agree). Self-reported height and weight for BMI. Internal consistency of the two adapted subscales was additionally assessed post hoc using Cronbach's alpha computed from the present dataset: alpha = 0.622 (healthy food perception subscale, A1-A7), alpha = 0.638 (food safety/hygiene subscale, B1-B7), and alpha = 0.729 for the combined 14-item instrument. |
| 9 | **Methods: Bias** | Describe efforts to address potential sources of bias | Discussion 4.1 / Limitations (p.11): social desirability bias, self-reported anthropometrics, convenience sampling, and dietary classification ambiguity are acknowledged with directional implications. A sixth limitation was added noting that the adapted instrument, although showing acceptable post hoc internal consistency (Section 2.3), had not undergone separate formal psychometric validation (e.g., confirmatory factor analysis, test-retest reliability) prior to fielding. |
| 10 | **Methods: Study size** | Explain how the study size was arrived at | Methods 2.2 (p.4): pre-specified minimum n=300 via WHO formula; achieved n=303. |
| 11 | **Methods: Quantitative variables** | Explain how quantitative variables were handled in analyses | Methods 2.4 (p.4): OFDA frequency dichotomized (frequent/infrequent) per prior validated approach. BMI was continuous and then categorized per WHO standards. Cuisine preferences dichotomized (healthy/unhealthy). |
| 12a | **Methods: Statistical methods** | Describe all statistical methods, including those used to control for confounding | Methods 2.5 (p.5): Shapiro-Wilk normality test; Levene test for variance equality; chi-square for categorical associations; Mann-Whitney U for 2-group comparisons; Kruskal-Wallis H for >=3 groups. |
| 12b |  | Describe methods used to examine subgroups and interactions | Methods 2.5 (p.5) and Results 3.3-3.4: subgroup analyses by sex, age, region, employment, and income. Post-hoc Dunn-Bonferroni correction applied after significant Kruskal-Wallis results. |
| 12c |  | Explain how missing data were addressed | Results 3.5 (p.7): BMI analysis n=302/303; one participant excluded for implausible anthropometric values. Supplementary Table S1 reports all n per analysis. |
| 12d* |  | Cross-sectional: analytical methods accounting for sampling strategy | N/A: convenience + snowball sampling not a complex probability design; no post-stratification weighting applied. Acknowledged as a limitation. |
| 12e |  | Describe any sensitivity analyses | N/A for this study. Noted as a future research direction. |
| 13a | **Results: Participants** | Report numbers at each stage of study | Methods 2.2 (p.4) and Supplementary Figure S1: 492 invited -> 166 declined -> 325 potential -> 22 excluded (ineligible) -> 303 final participants. |
| 13b |  | Give reasons for non-participation at each stage | Methods 2.2 (p.4): 166 declined to participate; 22 were excluded for never having used an OFDA. |
| 13c |  | Consider use of a flow diagram | Referenced as Supplementary Figure S1: Participant Flow Diagram. |
| 14a | **Results: Descriptive data** | Give characteristics of study participants (demographic, clinical, social) | Results 3.1 and Table 1 (p.6-7): full sociodemographic profile including sex, age, nationality, marital status, education, employment, region, living situation, income, and OFDA frequency distribution. |
| 14b |  | Indicate number of participants with missing data for each variable | Results 3.1-3.6: n=303 for all perception analyses; n=302 for BMI analysis; n=194 for healthy/unhealthy food choice analysis (mixed-cuisine responses excluded per pre-specified rationale). |
| 15* | **Results: Outcome data** | Report numbers of outcome events or summary measures | Results 3.2-3.6 and Tables 1-5: all outcomes reported as frequencies/percentages, means ± SD, medians, and exact p-values. |
| 16a | **Results: Main results** | Give unadjusted estimates and their precision (e.g., 95% CI) | Results 3.1-3.6: all results presented as unadjusted statistics (chi-square, Mann-Whitney U, Kruskal-Wallis H) with exact p-values. No multivariable regression was applied, given the descriptive/associative study objectives. |
| 16b |  | Report category boundaries when continuous variables were categorized | Methods 2.4 (p.4) and Results 3.5: BMI WHO cut-offs explicitly stated; OFDA frequency categorization defined and justified. |
| 16c |  | If relevant, translate estimates of relative risk into absolute risk | N/A: no relative risk calculations performed in this descriptive/associative study. |
| 17 | **Results: Other analyses** | Report other analyses done, e.g., subgroups and sensitivity analyses | Results 3.3-3.4 and Tables 2-3: subgroup analyses by sex, OFDA frequency, age, employment, region, and income reported systematically for all 14 perception items. |
| 18 | **Discussion: Key results** | Summarise key results with reference to study objectives | Discussion paragraph 1: six key findings summarized as numbered items (i)-(vi), each explicitly linked to the five stated objectives stated in the Introduction. |
| 19 | **Discussion: Limitations** | Discuss limitations, taking into account sources of potential bias or imprecision | Discussion 4.1 (p.11): cross-sectional design, convenience sampling, self-reported anthropometrics, social desirability, and dietary classification limitations are discussed with directional implications. A sixth limitation regarding the absence of formal psychometric validation of the adapted instrument prior to fielding was also added. |
| 20 | **Discussion: Interpretation** | Give cautious overall interpretation considering objectives, limitations, and other evidence | Discussion paragraphs 2-8 and Section 4.1 (Strengths and Limitations): each finding interpreted with reference to the most comparable published studies (Cheikh Ismail et al. 2024; Saleh et al. 2024; Eu and Sameeha 2021; Keeble et al. 2020); cautious language maintained; mechanistic explanations offered using 'may be attributed to' and 'may reflect' framing throughout. Following reviewer feedback, an explicit mechanistic ("why") explanation was added to each of the six main Discussion paragraphs, addressing regional/income disparities, income-related label responsiveness, choice-architecture effects on the intention-behavior gap, occupational health literacy, gender differences in food safety perception, and the null BMI association. A substantially expanded paragraph on regulatory and platform-design interventions (mandatory nutritional labelling, algorithmic promotion of healthy options, in-app hygiene rating integration, and price-equity mechanisms) was also added at the end of the Discussion, with two new supporting references (40, 41). |
| 21 | **Discussion: Generalisability** | Discuss the generalisability (external validity) of study results | Discussion 4.1 (p.11): generalizability limitations of young, female, Central-Province-dominant sample acknowledged; directions for more representative future research provided. |
| 22 | **Other: Funding** | Give the source of funding and role of funders | Funding section (p.12): Ongoing Research Funding program (ORF-2026-1554), King Saud University. Role: funding acquisition only; no involvement in study design, data collection, analysis, or reporting. |

*Note: Items marked * relate specifically to cross-sectional study design. N/A = Not applicable. This STROBE checklist is submitted as Supplementary Material S2.*
